# The secondary bile acids, ursodeoxycholic acid and lithocholic acid, protect against intestinal inflammation by inhibition of epithelial apoptosis

**DOI:** 10.14814/phy2.14456

**Published:** 2020-06-19

**Authors:** Natalia K. Lajczak‐McGinley, Emanule Porru, Ciara M. Fallon, Jessica Smyth, Caitriona Curley, Paul A. McCarron, Murtaza M. Tambuwala, Aldo Roda, Stephen J. Keely

**Affiliations:** ^1^ Department of Molecular Medicine Royal College of Surgeons in Ireland Beaumont Hospital Dublin 9 Ireland; ^2^ Department of Chemistry University of Bologna Bologna Italy; ^3^ School of Pharmacy and Pharmaceutical Sciences Ulster University Coleraine Northern Ireland; ^4^ INBB National Institute of Bio structures and Biosystems Rome Italy

**Keywords:** apoptosis, bile acid, colitis, epithelial barrier function

## Abstract

Increased epithelial permeability is a key feature of IBD pathogenesis and it has been proposed that agents which promote barrier function may be of therapeutic benefit. We have previously reported the secondary bile acid, ursodeoxycholic acid (UDCA), to be protective in a mouse model of colonic inflammation and that its bacterial metabolism is required for its beneficial effects. The current study aimed to compare the effects of UDCA, LCA, and a non‐metabolizable analog of UDCA, 6‐methyl‐UDCA (6‐MUDCA), on colonic barrier function and mucosal inflammation in a mouse model of colonic inflammation. Bile acids were administered daily to C57Bl6 mice by intraperitoneal injection. Colonic inflammation, induced by addition of DSS (2.5%) to the drinking water, was measured as disease activity index (DAI) and histological score. Epithelial permeability and apoptosis were assessed by measuring FITC‐dextran uptake and caspase‐3 cleavage, respectively. Cecal bile acids were measured by HPLC‐MS/MS. UDCA and LCA, but not 6‐MUDCA, were protective against DSS‐induced increases in epithelial permeability and colonic inflammation. Furthermore, UDCA and LCA inhibited colonic epithelial caspase‐3 cleavage both in DSS‐treated mice and in an in vitro model of cytokine‐induced epithelial injury. HPLC‐MS/MS analysis revealed UDCA administration to increase colonic LCA levels, whereas LCA administration did not alter UDCA levels. UDCA, and its primary metabolite, LCA, protect against intestinal inflammation in vivo, at least in part, by inhibition of epithelial apoptosis and promotion of barrier function. These data suggest that clinical trials of UDCA in IBD patients are warranted.

## Graphical abstract

Bacterial metabolism of UDCA to LCA protects against DSS‐induced colonic inflammation by preventing epithelial apoptosis and promoting barrier function. In conditions of IBD, increased production of proinflammatory cytokines and chemokines from the epithelium leads to recruitment of immune cells to the mucosa. Incoming immune cells produce additional cytokines and mediators that can promote epithelial apoptosis, leading to loss of barrier function. Administration of ursodeoxycholic acid (UDCA) leads to the generation of lithocholic acid (LCA) in the colon which, in turn, acts on the epithelium to inhibit cytokine‐induced apoptosis, thereby promoting barrier function and protecting against inflammation.

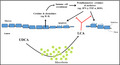

## 1 INTRODUCTION

Inflammatory bowel diseases, comprised mainly of ulcerative colitis, microscopic colitis, and Crohn's disease, are chronic and recurring disorders of the intestinal tract that are characterized by inflammation of the intestinal wall. Collectively, IBDs affect 1%–2% of the population in Western societies, although the incidence is rapidly increasing (Windsor & Kaplan, 2019). While the pathogenesis of IBD is still not well understood, it is accepted that a complex interplay of environmental and genetic factors is involved. Depending on the disease severity, symptoms vary from abdominal pain to severe diarrhea, ulceration and intestinal bleeding, malnutrition, anxiety, and depression. Current treatments for IBD make use of a range of anti‐inflammatory drugs, including aminosalicylates, glucocorticoids, immunosuppressants, and biologics, with these drugs primarily targeting the mucosal immune system to reduce the inflammatory cell influx and dampen the production of cytokines, chemokines, and pro‐inflammatory chemical mediators (Seyedian, Nokhostin, & Malamir, 2019). While these drugs can be effective in inducing or maintaining remission in patients, their clinical utility is often limited by lack of efficacy, development of drug‐resistance, or the occurrence of serious side effects and new therapeutic options are still required.

The pathogenesis of IBD is closely associated with increased epithelial permeability to luminal macromolecules (Vancamelbeke & Vermeire, 2017). Whether such loss of epithelial barrier function is a cause or consequence of disease is still uncertain but studies, showing that intestinal permeability is increased in disease‐free first‐degree relatives and spouses of patients, suggest it is likely to be a predisposing factor (Soderholm et al., 1999). Epithelial barrier function is a complex and multifaceted entity comprised of both physical and biochemical components and the factors causing its disruption in IBD are still not well understood. However, it is well established that central to the maintenance of the barrier is appropriate regulation of epithelial cell turnover by apoptosis (Camilleri, 2019). Apoptosis, or programmed cell death, is a process that occurs normally as differentiating epithelial cells migrate from the crypts to the villus tips (or surface of the colon) and are shed into the lumen. An appropriate balance between proliferation in the crypts and apoptosis at the villus tip/surface is essential to maintaining continuity to the epithelial barrier. In conditions of IBD, there is an upregulation of epithelial apoptosis leading to disruption of the physical barrier that these cells pose at the interface with the luminal contents (Michielan & D'Inca, 2015). In turn, barrier dysfunction leads to increased permeability to luminal microbes, toxins, and allergens, thereby promoting immune cell infiltration and activation and amplifying the inflammatory response. Given the importance of increased apoptosis and dysregulated epithelial barrier function in the pathogenesis of IBD, there is a great deal of interest in its targeting for disease treatment. However, to date, there are still no therapeutic options which act by directly targeting the epithelium to prevent apoptosis in conditions of IBD.

There has long been an association between altered levels of luminal bile acids and the pathogenesis of IBD (Tiratterra et al., 2018), and recent studies suggest that bile acids are likely to have a role to play in the enhanced epithelial permeability that is associated with disease progression (Munch, Strom, & Soderholm, 2007; Stenman, Holma, & Korpela, 2012). However, unlike other dihydroxy bile acids present in the colon, ursodeoxycholic acid (UDCA) has long been known to have therapeutic properties. UDCA, as the bioactive component of bear bile, has long been used in Traditional Chinese Medicine to treat a wide range of illnesses and more recently chemically synthesized UDCA has been used in Western medicine to treat cholestatic liver diseases, such as gallstones and primary sclerosing cholangitis (Cabrera, Arab, & Arrese, 2019; Wang & Carey, 2014). The therapeutic properties of UDCA in the liver have been, at least partly, ascribed to its capacity to prevent apoptosis (Amaral, Viana, Ramalho, Steer, & Rodrigues, 2009; Beuers, 2006). Indeed, based on its potent cytoprotective actions, UDCA is currently under investigation for a range of diseases that are associated with increased apoptotic cell death, including neurological, ocular, and cardiovascular disorders (Vang, Longley, Steer, & Low, 2014).

Several studies have shown that UDCA and its taurine conjugate (TUDCA) are protective in preclinical models of intestinal inflammation (Kullmann, Arndt, Gross, Ruschoff, & Scholmerich, 1997; Laukens et al., 2014; Martinez‐Moya et al., 2013; Van den Bossche et al., 2017). In the DSS model of intestinal disease, where inflammation is initiated due to a breakdown of epithelial barrier function (Eichele & Kharbanda, 2017), we have also demonstrated UDCA to reduce disease severity and to dampen mucosal cytokine expression. (Ward et al., 2017). Furthermore, since a metabolically stable analogue of UDCA, 6‐methyl‐UDCA (6‐MUDCA) was without protective effects, we proposed that bacterial metabolism of UDCA was necessary for the full expression of its beneficial actions. In keeping with this, we found that administration of the primary colonic metabolite of UDCA, lithocholic acid (LCA), was even more potent than its parent compound in protecting mice from DSS‐induced colonic inflammation. Here, we set out to build on our previous work by directly comparing the effects of UDCA and LCA in the DSS model of colonic inflammation and to investigate whether these bile acids exert their beneficial actions by directly targeting cytokine‐induced epithelial apoptosis and barrier function.

## 2 MATERIALS AND METHODS

### 2.1 Animal studies

Ethical approval for experiments carried out on mice was obtained from the RCSI Ethics Committee and the Health Products Regulatory Authority of Ireland (HPRA; Authorization #: AE19127/P047). Male C57Bl/6 mice were used between 10 and 12 weeks of age. Colitis was induced in mice by addition of 2.5%DSS (MP Biomedicals, Solon, OH) to the drinking water for 6 days. Disease activity index (DAI) was used as a measure of disease progression and was calculated by the addition of scores designated to body weight, fecal blood, and stool consistency/diarrhea, as previously described (Tambuwala et al., 2015). Starting one day prior to the administration of DSS, animals received daily intraperitoneal injections of either endotoxin‐free PBS (vehicle control), Na^+^‐UDCA (30 mg/kg), Na^+^‐LCA (30 mg/kg), or Na^+^‐6α‐MUDCA (30 mg/kg) dissolved in PBS. On day 6, mice were administered FITC‐dextran (6 mg/kg) by oral gavage. Mice were sacrificed on day 7 and their colons were removed and measured. Cecal contents were collected, mixed with an equal volume of isopropanol, and stored until analysis for bile acid content. For histological scoring, approximately 1 cm sections of distal colonic tissue were fixed in 10% paraformaldehyde (pH 7.4; PBS buffered) and embedded in paraffin. Sections (4 µm) were cut, stained with H&E, and blindly examined for histological score by 2 independent observers, as previously described (Tambuwala et al., 2015). Staining for cleaved caspase‐3 in epithelial cells of colonic sections was carried out using anti‐cleaved caspase‐3 (Asp175) (5A1E) (Cell Signalling Technologies). Quantification of epithelial cells stained for cleaved caspase‐3 was assessed in a blinded fashion using Aperio Scanscope software by two independent observers.

For measurements of MPO activity, sections of distal colonic mucosa were stripped of their smooth muscle and epithelial layers, homogenized on dry ice, and assayed using a kit according to the manufacturer's instructions (Sigma‐Aldrich). For measurements of FITC‐dextran uptake from the lumen, blood was collected by cardiac puncture and centrifuged at 1,500*g* (15 min, 4°C). Levels of FITC‐dextran in the serum were then measured fluorometrically, according to the manufacturer's instructions (Sigma‐Aldrich).

### 2.2 Cell culture and treatments

T_84_ colonic epithelial cells were grown in Dulbecco's modified Eagle's medium (DMEM)–Ham's F12 nutrient mixture (1:1), supplemented with 5% fetal bovine serum (FBS), 1% penicillin/streptomycin, and 1% L‐Glutamine (Gibco). For measurements of FITC‐dextran flux, cells were seeded onto 12 mm Milicel‐HA Transwell inserts (Milipore, Merck) at a density of 5 × 10^5^ cells/insert. For western blot analyses, cells were seeded onto 24 mm Millicell‐HA cell culture inserts at a density of 2 × 10^6^ cells/insert. Cells were cultured on inserts until they attained an electrically resistant phenotype, that is, when transepithelial resistance (TEER) reached approximately 1 KΩ/cm^2^, as measured using an EVOM2™ Voltohmmeter (World Precision Instruments).

### 2.3 Measurements of FITC‐dextran flux

Cells were washed and incubated in serum‐free medium for 1 hr prior to treatment with IFNγ (40 ng/ml, Peprotech) for 24 hr. Cells were then treated with TNF (20 ng/ml, Peprotech) in combination with either UDCA (100 µM), LCA (10 µM), or 6‐MUDCA (100 µM). Simultaneously, 5 μl of FITC‐dextran (10 mg/ml) was added to the apical side of the Transwell insert. After a further incubation of 24 hr, medium from the basolateral side of the cell monolayers was collected and fluorescence intensity was measured on a Victor X3 plate reader (Perkin Elmer) set to 485 nm excitation and 520 nm emission wavelengths.

### 2.4 Western blotting

Treated monolayers of T_84_ cells were scraped from their inserts and homogenized in lysis buffer (130 mM glycine, 2% sodium dodecyl sulphate [SDS], 7.7% glycerol in 70 mM Tris‐HCl, pH 8.8) by repeated passage through a 26‐gauge needle. Samples, normalized for protein content, were mixed with an equal volume of 2 × laemmli loading buffer (1/1, v/v) (Sigma), boiled for 5 min, and loaded onto a 8% SDS‐tricine polyacrylamide gel. After electrophoresis, transfer to PVDF membranes (Millipore) was performed for 2 hr at 0.15 A in 0.05 M sodium borate solution, pH 9.0, with 20% methanol and 0.05% SDS. Immunoblotting was performed with antibodies against cleaved‐PARP (Catalogue #: 9,546; Cell Signalling Technology). Cleaved‐PARP levels were quantified by densitometry (ImageQuantTLInk software) and normalized to β‐actin as a protein loading control (Abcam).

### 2.5 Cecal bile acid analysis

Cecal bile acid levels were identified and quantified by high‐pressure liquid chromatography‐electrospray‐mass spectrometry/mass spectrometry (HPLC‐ES‐MS/MS) by recent published method suitable for use in pure standard solution, intestinal content, and stool samples after appropriate pre‐analytical procedures. Liquid chromatography analysis was performed using an Alliance HPLC system model 2695 from Waters combined with a triple quadruple mass spectrometer QUATTRO‐LC (Micromass; Waters) using an electrospray interface. The analytical column was a Waters XSelect CSH C18 column, 5 µm, 150 × 2.1 mm, protected by a self‐guard column Waters XSelect CSH C18 5 µm, 10 × 2.1 mm. BAs were separated by elution gradient mode with a mobile phase composed of a mixture ammonium acetate buffer 15 mM, pH 8.0 (Solvent A) and methanol (Solvent B). Chromatograms were acquired using the mass spectrometer in multiple reaction monitoring mode. Briefly, aliquots of cecal sample homogenate (0.3 g) were extracted with 0.9 ml of isopropanol. The mixture was stirred for 30 min at 37°C, then centrifuged at 800 g for 5 min. The supernatant was then diluted 1:10 (v/v) with 40% isopropanol in 15 mM ammonium acetate at pH 8.00, filtered, transferred to an autosampler vial, and 5 μl injected into the HPLC‐ESI‐MS system.

### 2.6 Statistical analysis

Results are expressed as mean ± *SEM* for a series of *n* experiments. Statistical analyses were performed by ANOVA with the Tukey multiple comparisons post‐test using GraphPad Instat software (GraphPad). *p* values ≤.05 were considered to be statistically significant.

## 3 RESULTS

### 3.1 Analysis of cecal bile acids in mice administered UDCA, LCA, or 6‐MUDCA

**FIGURE 1:**
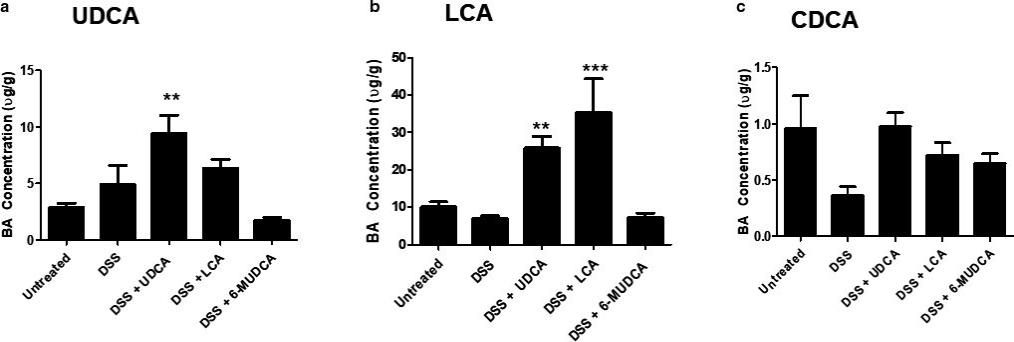
Analysis of cecal bile acids in mice administered UDCA, LCA, or 6‐MUDCA. Starting 24 hr prior to administration of DSS (2.5% in the drinking water), and daily thereafter, separate groups of male C57BL6 mice received endotoxin‐free PBS or Na^+^‐UDCA (30 mg/kg), Na^+^‐LCA (30 mg/kg), or Na^+^‐6α‐MUDCA (30 mg/kg) dissolved in PBS by IP injection. On the 7th day, mice were sacrificed and the cecal content was collected and analyzed for levels of (a) UDCA, (b) LCA, and (c) CDCA, as described in Materials and Methods. Data are expressed as mean ± sem. *n* = 12; **p < .01, *** *p* < .001 compared to untreated controls

We first analyzed levels of UDCA and LCA in the cecal water before and after treatments with the bile acids. Under basal conditions, LCA was more prevalent than UDCA at 10.1 ± 1.3 and 2.9 ± 1.4 µg/ml, respectively (*n* = 12). Treatment with DSS tended to increase the levels of UDCA and decrease those of LCA but these effects were not statistically significant. Daily administration of UDCA significantly increased cecal UDCA levels to 9.4 ± 1.6 µg/ml (*n* = 12; *p* < .01) and LCA levels to 25.9 ± 3.0 µg/ml (*n* = 12; *p* < .01). Administration of LCA to the mice did not a significantly alter cecal UDCA concentrations but increased LCA levels to 35.4 ± 9.0 µg/ml (*p* < .001; *n* = 12). Under basal conditions, the primary bile acid, CDCA, was present at relatively low amounts (1.0 ± 0.3 µg/mg; *n* = 12). None of the treatments used significantly altered CDCA levels although DSS tended to decrease levels of the bile acid. Treatment of the mice with the non‐metabolizable UDCA analog, 6‐MUDCA, did not alter levels of LCA or UDCA (Figure 1).

### 3.2 UDCA and LCA, but not 6‐MUDCA, protect against DSS‐induced intestinal inflammation in mice

**FIGURE 2:**
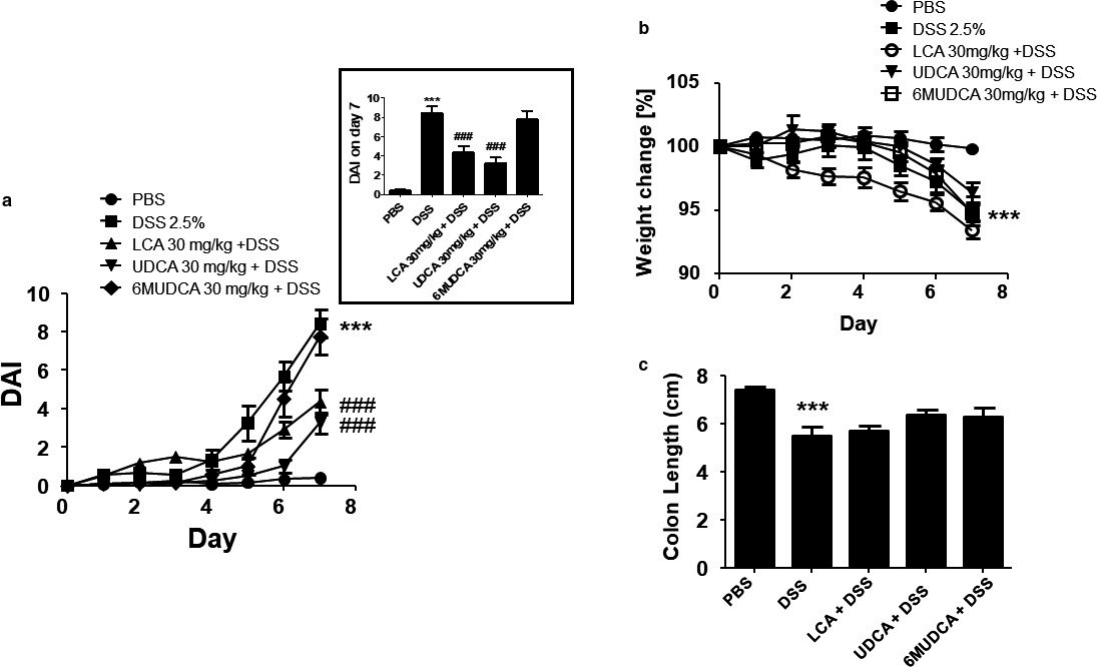
Effects of UDCA, LCA, and 6‐MUDCA on disease activity in DSS‐treated mice. Starting 24 hr prior to administration of DSS (2.5% in the drinking water), and daily thereafter, separate groups of male C57BL6 mice received either endotoxin‐free PBS or Na^+^‐UDCA (30 mg/kg), Na^+^‐LCA (30 mg/kg), or Na^+^‐6α‐MUDCA (30 mg/kg) dissolved in PBS by IP injection. (a) Disease activity index (DAI) was assessed daily (*n* = 12). For clarity, the inset depicts DAI at the end of the treatment period on Day 7. (b) Body weight was assessed daily to monitor disease progression. (c) Mice were sacrificed on day 7 and their colons were removed and measured (*n* = 5–6). ***p < .001 compared to controls (no DSS treatment); ^##^ *p* < .001 compared to DSS‐treated mice

Administration of DSS (2.5%) in the drinking water increased DAI to 8.4 ± 0.7 by day 7 of the study (Figure 2a). This was accompanied by significant weight loss and shortening of the colon (Figure 2b and c). Daily treatment with either UDCA or LCA significantly attenuated DSS‐induced disease activity to 3.3 ± 0.6 and 4.3 ± 0.7, respectively. However, administration of 6‐MUDCA was without effect. None of the bile acids significantly altered DSS‐induced weight loss although there was a tendency for UDCA to reduce and LCA to increase weight loss. None of the bile acids tested significantly altered colonic shortening in response to DSS.

**FIGURE 3:**
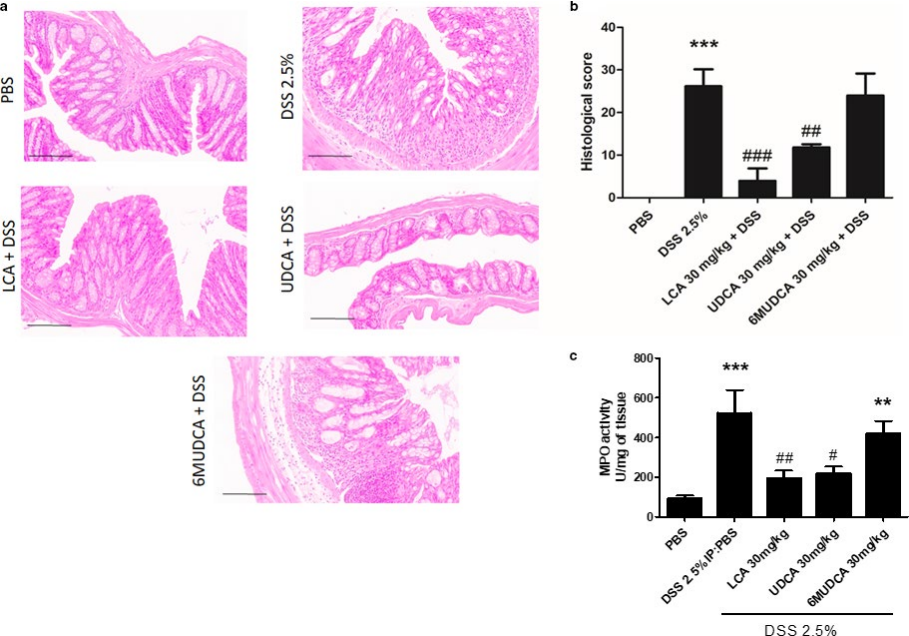
Effects of UDCA, LCA, and 6‐MUDCA mucosal inflammation in DSS‐treated mice. Starting 24 hr prior to administration of DSS (2.5% in the drinking water), and daily thereafter, separate groups of male C57BL6 mice received endotoxin‐free PBS or Na^+^‐UDCA (30 mg/kg), Na^+^‐LCA (30 mg/kg), or Na^+^‐6α‐MUDCA (30 mg/kg) dissolved in PBS by IP injection. (a) Sections of colon from control, DSS‐treated and bile acid‐treated C57BL6 mice were taken and processed for H&E staining. (b) Inflammation score was assessed as described in Materials and Methods (*n* = 3). (c) Sections of distal colon were stripped of their muscle layers and MPO activity was assessed (*n* = 5–6); **p < .01,***p < .001 compared to controls (no DSS treatment); ^#^ *p* < 005, ^##^ *p* < .01, ^###^ *p* < .001 compared to DSS‐treated mice

Histological evaluation of the distal colon revealed that treatment with DSS increased inflammatory cell infiltration, mucosal edema, and epithelial damage leads to an overall increase in the histological inflammation score (Figure 3a and b). However, in mice treated with UDCA, the histological score was significantly reduced from 26.2 ± 4.0 in mice treated with DSS alone to 11.8 ± 0.7 (*p* < .01; *n* = 12). Furthermore, in mice treated with LCA, the inflammation score was reduced by an even greater extent to 3.9 ± 2.9 (*p* < .001; *n* = 12). In contrast, 6‐MUDCA treatment did not significantly alter the DSS‐induced increase in histological inflammation score. As a biochemical index of neutrophil influx to the mucosa, we also measured mucosal myeloperoxidase levels. Upon DSS treatment, MPO levels increased in the mucosa and this effect was significantly reduced by treatment with either UDCA or LCA, but not with 6‐MUDCA (Figure 3c).

### 3.3 UDCA and LCA, but not 6‐MUDCA, inhibit DSS‐induced epithelial apoptosis increased mucosal permeability in vivo

**FIGURE 4:**
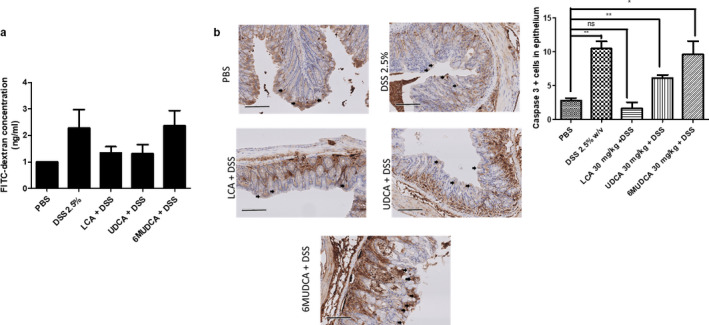
Effects of UDCA, LCA, and 6‐MUDCA on barrier function and epithelial caspase‐3 cleavage in DSS‐treated mice. Starting 24 hr prior to administration of DSS (2.5% in the drinking water), and daily thereafter, separate groups of male C57BL6 mice received endotoxin‐free PBS, Na^+^‐UDCA (30 mg/kg), Na^+^‐LCA (30 mg/kg), or Na^+^‐6α‐MUDCA (30 mg/kg) by IP injection. On the 6th day, mice were administered FITC‐dextran (6 mg/kg) by oral gavage and after a further 24 hr mice were sacrificed. (a) Levels of FITC‐dextran in serum samples were measured (*n* = 12). (b) Sections of colon from control, DSS‐treated and DSS + bile acid‐treated C57BL6 mice were taken and processed for immunohistochemical staining with Cleaved Caspase‐3 antibody (*n* = 3) *p < .05, ***p < .001 compared between indicated groups

Next, we went on to investigate whether the protective effects of UDCA and LCA against the onset of colonic inflammation might be due to a restoration of epithelial barrier function. To assess epithelial permeability, we employed the fluorescent marker, FITC‐dextran, to measure its appearance in the blood after oral administration. Induction of colonic inflammation with DSS increased the appearance of FITC‐dextran the blood, indicating enhanced epithelial permeability to the macromolecule. However, treatment of the mice with either UDCA or LCA abolished this effect. In contrast, in 6‐MUDCA‐treated mice, levels of FITC‐dextran in the blood were similar to those in mice treated with DSS alone (Figure 4a).

Given the importance of regulated cell death in the maintenance of intestinal barrier function, we assessed the effects of bile acid treatment on DSS‐induced apoptosis of the epithelial layer. For these experiments, we analysed the levels of the apoptotic marker, cleaved caspase‐3, in the epithelium of colonic sections. As expected, administration of DSS increased the levels of cleaved caspase‐3 in the colonic epithelium, indicating increased apoptosis. However, this effect was significantly attenuated when mice were treated with either UDCA or LCA. In contrast, treatment with 6‐MUDCA failed to prevent DSS‐induced caspase‐3 cleavage (Figure 4b and c).

### 3.4 UDCA and LCA, but not 6‐MUDCA, inhibit inflammatory cytokine‐induced increases in colonic epithelial apoptosis and permeability in vitro

**FIGURE 5:**
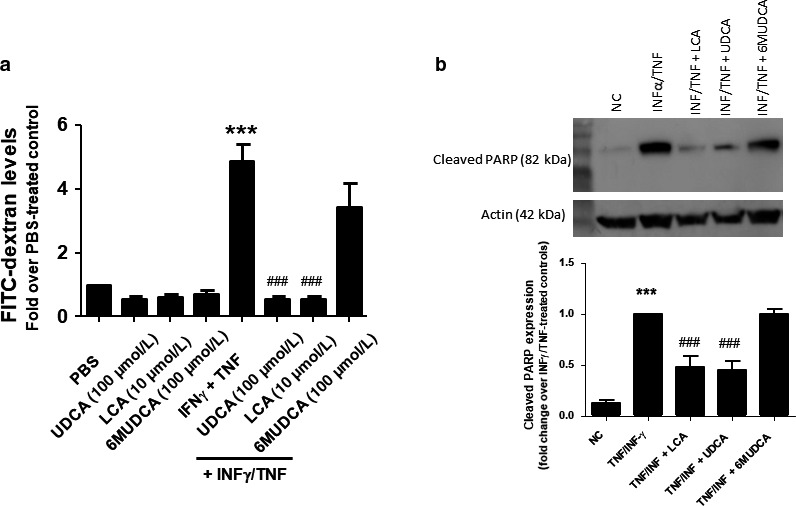
Effects of UDCA, LCA, and 6‐MUDCA on colonic epithelial barrier function in vitro. Monolayers of T_84_ cells grown on permeable supports were treated with IFN‐γ (40 ng/ml) 24 hr prior adding TNF (20 ng/ml) alone or in combination with Na^+^‐UDCA (100 µM), Na^+^‐LCA (10 µM) or Na^+^‐6α‐MUDCA (100 µM). FITC‐dextran (10 mg/ml) was then added to the apical side. (a) After 24 hr, the basolateral media was collected and FITC‐dextran levels were measured (*n* = 4). (b) Lysates from cells treated with IFNγ and TNF in the absence or presence of UDCA, LCA, or 6‐MUDCA were analyzed for levels of the p89 fragment of cleaved PARP by western blotting. Panel c shows densitometric analysis of 5 similar experiments. ***p < .05 compared to untreated cells; ^###^ *p* < .001 compared to TNF/IFNγ‐treated cells.

Finally, to investigate whether the protective effects of UDCA and LCA might be due to direct actions on the epithelial cells themselves, we employed the T_84_ colonic epithelial cell line. When grown as on permeable supports, these cells form electrically resistant monolayers that provide a well‐established reductionist model of colonic epithelial barrier function. To mimic the inflammatory environment in vivo, cells were treated with the pro‐inflammatory cytokines, TNF‐α, and IFN‐γ. Under these conditions, cytokine‐treated cells underwent increased apoptosis, as indicated increased levels of cleaved PARP by Western blotting, and this was associated with significantly increased permeability of the monolayers to FITC‐dextran (Figure 5). However, in cells treated with either UDCA or LCA, cytokine‐induced increases in apoptosis and FITC‐dextran permeability were abolished, whereas 6‐MUDCA was without significant effect.

## 4 DISCUSSION

As we enter into the third decade of the 20th century, the incidence of IBD continues to increase around the world, with Western populations being particularly susceptible (Windsor & Kaplan, 2019). While we have learned much about the genetic and environmental factors underlying pathogenesis of the disease, they are still not fully defined and our understanding of the cellular and molecular mechanisms involved is still incomplete. Consequently, despite intensive research efforts, the development of new approaches for IBD treatment has been lacking. The first line of treatment continues to be immunosuppressants, the use of which in many patients is limited by lack of efficacy, development of resistance, and the occurrence of adverse effects. New lines of treatment are still urgently required and there has been growing interest in the potential for targeting epithelial barrier function for this purpose. Increased permeability of the epithelial layer to luminal macromolecules has long been appreciated as an early event in the pathogenesis of disease in both animal models and humans (Chassaing, Aitken, Malleshappa, & Vijay‐Kumar, 2014; Kitajima, Takuma, & Morimoto, 1999). Indeed, the observations that abnormalities in colonic barrier function exist in disease‐free first‐degree relatives of IBD patients suggest that it may be a fundamental factor in disease onset (Soderholm et al., 1999). With this in mind, it is thought that drugs which target the epithelium to promote barrier function may provide an effective alternative to currently available treatments (Camilleri, 2019). The current studies suggest that the bile acid, UDCA, has potential to be developed for use in such a way.

As we and others have previously reported, we found UDCA to exert protective actions against DSS‐induced colonic inflammation in mice, a model considered to be particularly suitable for studying the role of epithelial barrier function in disease pathogenesis (Eichele & Kharbanda, 2017). After 7 days of DSS treatment, administration of UDCA decreased disease activity index by approximately 50% and this was accompanied by a similar attenuation of the histological inflammation score. Treatment with UDCA prevented epithelial damage and this was accompanied by a functionally restored barrier, as demonstrated by reduced flux of FITC‐dextran from the lumen into the blood after its oral administration. This barrier‐promoting effect of UDCA was associated with reduced influx of granulocytes into the mucosa, as determined histologically by H&E staining and biochemically, by measurements of mucosal MPO levels. Thus, findings from the current studies confirm our previous observations and support a growing body of evidence to suggest that UDCA may have a role to play in the treatment of intestinal inflammatory diseases.

In the current study, we have extended our previous work to directly compare the effects of UDCA, with that of its primary metabolite, LCA, and a non‐metabolizable analog, 6‐MUDCA, on inflammation and barrier function in the DSS model while measuring colonic levels of these bile acids after treatment. We found that similar to UDCA, treatment of the mice with LCA attenuated DSS‐induced disease activity and was even more effective in reducing the histological inflammation score and mucosal levels of MPO. LCA also prevented the DSS‐induced increase in mucosal permeability to FITC‐dextran, demonstrating enhanced epithelial barrier function in response to treatment with the bile acid. In contrast, 6‐MUDCA was without significant effect on either disease activity, histological inflammation score, mucosal MPO levels, or FITC‐dextran uptake into the blood. Thus, our current data confirm previous findings that bacterial metabolism of UDCA is necessary for the expression of its anti‐inflammatory actions in vivo. This is further supported by our analysis of cecal bile acid levels in mice treated with UDCA, LCA, or 6‐MUDCA. In 6‐MUDCA‐treated mice, levels of UDCA and LCA were unchanged and protective effects against DSS‐induced inflammation were not observed. In UDCA‐treated mice, where protective actions were observed, both UDCA and LCA levels were significantly elevated in the colon. Similarly, protective effects were apparent in LCA‐treated mice, where only LCA and not UDCA levels were elevated in the cecal content. Thus, it appears to be the presence of elevated levels of LCA in the colon that is most closely associated with protection against colonic inflammation in this model.

Intestinal barrier function is a complex entity comprised of several physical and biochemical factors, most of which appear to be disrupted in conditions of IBD (Vancamelbeke & Vermeire, 2017). Increased epithelial cell death by apoptosis is recognized to play an important role in enhanced mucosal permeability, both in animal models and human patients (Gunther, Buchen, Neurath, & Becker, 2014; Seidelin, 2015). The data presented here demonstrate that treatment with UDCA inhibits colonic epithelial apoptosis, both in vitro and in vivo. The idea that UDCA can exert anti‐apoptotic actions is not new, with previous studies demonstrating its cytoprotective actions in hepatocytes (Sommerfeld, Reinehr, & Haussinger, 2015; Tsagarakis, Drygiannakis, Batistakis, Kolios, & Kouroumalis, 2010). To date, there have been few studies of the effects of UDCA on apoptosis in enterocytes, with these focussing primarily on the protective effects of the bile acid in preventing apoptotic responses to deoxycholic acid (Im, Akare, Powell, & Martinez, 2005; Saeki et al., 2012). Only one previous study has investigated the actions of the taurine‐conjugated derivative of UDCA, TUDCA, on cytokine‐induced apoptosis in colonic epithelial cells where, similar to the current studies, it was found to be protective (Laukens et al., 2014). However, very high concentrations of TUDCA (>5 mM) were required for protective effects to be observed, with the authors suggesting that, due to lack of uptake mechanisms for luminal bile acids in the colon, entry of hydrophilic TUDCA into the cell likely only occurs only when its critical micellar concentration (CMC) is exceeded. The current study is the first to show that UDCA, at levels well below its CMC, can enter the colonic epithelium to directly inhibit cytokine‐induced apoptosis. Thus, in the setting of colitis, increased barrier function in response to UDCA administration would be expected to prevent the entry of bacteria and their toxins into the mucosa, thereby alleviating inflammation. It should be noted that the current study does not preclude that the barrier‐promoting effects of UDCA and LCA may not only be due to inhibition of apoptosis but may also involve the preservation of tight junction integrity. Epithelial tight junctions are known to become dysregulated, with increased permeability to macromolecules, in conditions of inflammation and future studies should be conducted to assess how UDCA and LCA may impact this.

Since, 6‐MUDCA was without effect on colonic epithelial apoptosis in vivo, while LCA had similar protective actions to UDCA, our data support a model in which bacterial conversion of UDCA to LCA is necessary for it to exert anti‐apoptotic actions. These data complement our previous studies in which we found that LCA also potently inhibits the release of pro‐inflammatory cytokines from the colonic epithelium (Ward et al., 2017), and contradict the classical view of LCA being a “toxic” bile acid. While it is clear that when present at high concentrations, LCA can induce epithelial apoptosis (Katona, Anant, Covey, & Stenson, 2009), its role in regulating barrier function at lower concentrations needs to be reconsidered. Interestingly, previous studies have reported that colonic LCA levels are suppressed in DSS‐treated mice (Araki, Andoh, Tsujikawa, Fujiyama, & Bamba, 2001; Van den Bossche et al., 2017), suggesting the bile acid may have a role to play in dampening the inflammatory response. Furthermore, levels of Clostridial bacterial strains that possess the 7‐β‐hydroxylase enzymatic activity required to convert UDCA to LCA have been shown to be decreased in patients with IBD (Paramsothy et al., 2019). A protective role for LCA is further supported by two recent studies in a canine model of chronic inflammatory enteropathy, where colonic inflammation was associated with decreased LCA levels and diet‐ or steroid‐induced remission in this model was associated with increased stool LCA, but not UDCA, levels (Guard et al., 2019; Wang et al., 2019). Such a reciprocal relationship between LCA levels and the degree of mucosal injury suggests a role for the bile acid as an endogenous suppressor gut inflammatory responses.

In conclusion, UDCA administration prevents cytokine‐induced epithelial apoptosis, promotes barrier function, and is protective in a mouse model of colonic inflammation. Bacterial metabolism of UDCA to LCA is required for such beneficial effects to be apparent. The molecular mechanisms by which LCA mediates its anti‐apoptotic and anti‐inflammatory actions remain to be identified and are the subject of ongoing work in our laboratory. While several possibilities exist, it is likely that LCA acts through one of the bile acid receptors known to be present in the colonic epithelium including, FXR, TGR5, and VDR. Each of these receptors can be activated by LCA (Alemi et al., 2013; Ishizawa, Akagi, & Makishima, 2018; Zimber & Gespach, 2008), and each has been shown to exert protective actions in mouse models of colonic inflammation (Gadaleta et al., 2011; Ren et al., 2019; Wang et al., 2017). These receptors, along with therapeutic strategies that appropriately modulate UDCA/LCA levels in the colon, represent excellent opportunities for the development of new approaches to promote barrier function and prevent inflammation in patients with IBD.

## CONFLICT OF INTEREST

None declared.

## AUTHORS’ CONTRIBUTIONS

Natalia K. Lajczak‐Mc Ginley: designed and performed the experiments, derived the models and analyzed the data. Emanule Porru, Aldo Roda: performed the bile acid analysis. Ciara M. Fallon, Jessica Smyth, and Caitriona Curley: assisted with animal experiments. Paul A. McCarron and Murtaza M. Tambuwala: provided histological scoring and immunohistological measurement. Stephen J. Keely: secured the funding, designed the experiments, and wrote the manuscript.
